# Supplementary material for: The promotion effect of π-π interactions in Pd NPs catalysed selective hydrogenation
Source: Nat Commun. 2022 Apr 1;13:1770. doi: 10.1038/s41467-022-29299-0 (PMC8975908; doi:10.1038/s41467-022-29299-0)
Supplement: Supplementary file 1 — Supplementary Information [file 41467_2022_29299_MOESM1_ESM.pdf]

The promotion effect of π-π interactions in Pd NPs catalysed selective hydrogenation

Miao Guo[1#], Sanjeevi Jayakumar[1#], Mengfei Luo[2*], Xiangtao Kong[3], Chunzhi Li[1,4], He Li[1], Jian Chen[2], & Qihua Yang[1, 2*]

[1] State Key Laboratory of Catalysis, Dalian Institute of Chemical Physics, Chinese Academy of Sciences, Dalian 116023, China.

[2] Key Laboratory of the Ministry of Education for Advanced Catalysis Materials, Zhejiang Key Laboratory for Reactive Chemistry on Solid Surfaces, Institute of Physical Chemistry, Zhejiang Normal University, Jinhua 321004, China.

[3] College of Chemistry and Chemical Engineering, Anyang Normal University, Anyang 455000, China.

[4] University of Chinese Academy of Sciences, Beijing 100039, China.

[#] These authors contributed equally to this work.

*E-mail: mengfeiluo@zjnu.cn, yangqh@dicp.ac.cn.

KEYWORDS: Heterogeneous hydrogenation, COFs, Pd NPs, Acetophenone, Pyrene, Weak interactions.

Supplementary methods

Chemicals

All materials were of analytical grade and used as received without any further purification. 1, 3 ,5-tris(p-formylphenyl)benzene (TFPB) was purchased from Jilin Chinese Academy of Sciences-Yanshen Technology Co., Ltd. 1, 3, 5-tris(4-aminophenyl)benzene (TAPB) and Na_2PdCl_4 were obtained from Innnochem Chemcals (Beijing). 1, 3, 6, 8-tetrakis(4-aminophenyl)pyrene (Py) and 2,5-dimethoxytere phthalaldehyde (DMTA) were synthesized according to the literature methods[1, 2]. The other reagents were purchased from Shanghai Chemical Reagent, Inc., of the Chinese Medicine Group.

Material characterization

N_2 sorption isotherms were performed on a Micromeritics ASAP 2020 system volumetric adsorption analyser. The Brunauer–Emmett–Teller (BET) surface area was calculated from the adsorption data at a relative pressure P/P_0 in the range of 0.04–0.20. Pore size distributions were determined from a nonlocal density functional theory (NLDFT) method. FT-IR spectra in the range of 400 to 4000 cm^{-1} were collected with a Nicolet Nexus 470 IR spectrometer using KBr pellets. Solid-state ^{13}C cross polarization/total sideband suppression (^{13}C-CP/TOSS) spectra were recorded on a Bruker 600 MHz spectrometer. C, H, N elemental analysis was performed with an O/N/H Analyzer (EMGA-930) and a C/S Analyzer (EMIA-8100). To reduce the N_2 contamination (from adsorbed air) during the N analysis process, we used mixture of Py-COF and KBr instead of pure Py-COF. The thermogravimetric analysis (TGA)

was performed using a NETZSCH STA 449F3 analyser from 30 to 900 °C with a heating rate of 5 °C min^{-1} under air atmosphere. Transmission electron microscopy (TEM) was performed using a Hitachi HT7700 at an accelerating voltage of 100 kV. High-resolution transmission electron microscopy (HRTEM) and energy-dispersive X-ray spectrometer (EDS) were obtained using a JEOL F200 instrument. Powder X-ray diffraction (PXRD) patterns of samples were measured on a Rigaku RINT D/Max-2500 powder diffraction system operated at 40 kV and 200 mA using Cu K_α radiation (a scan rate of 5° min^{-1}). X-ray photoelectron spectroscopy (XPS) was recorded on a VG ESCALAB MK2 apparatus using Al K_α (h_λ = 1486.6 eV) as the excitation light source. The results were calibrated by setting the C_{1s} adventitious carbon peak position to 284.6 eV. The metal contents of the samples were determined using a PLASAM-SPEC-II inductively coupled plasma atomic emission spectrometer (ICP) by digesting the COFs in HNO_3/H_2SO_4 (1: 1, v/v).

In situ FT-IR of CO adsorption. The in situ FT-IR spectra of CO adsorption were collected on a Bruker EQUINOX 55 infrared spectrometer with a MCT detector. Before the measurement, the catalyst was pre-treated at 200 °C under a flowing H_2 atmosphere (20 mL min^{-1}) for 1 h. The catalyst was subsequently cooled to room temperature. After the system was purged with Ar for 30 min, the background spectrum was collected. CO adsorption experiments were carried out by collecting 64 scans at a resolution of 4 cm^{-1}. Gas-phase CO spectra were collected at the same pressure and subtracted from the corresponding sample spectra.

CO chemisorption experiments. CO chemisorption measurement was performed at

50 °C on an Autochem II 2920 chemisorption instrument with a thermal conductivity detector (TCD). For CO chemisorption, the sample (~50 mg) was pre-treated with hydrogen at desired temperatures for 1 h, followed by purging with high-purity He for 30 min. After cooling down to 50 °C, a 5% CO/He mixture was injected into the reactor repeatedly until CO adsorption was saturated. The dispersion of Pd was calculated from the adsorbed amount of CO by assuming the CO/Pd adsorption stoichiometry to be 1/2 (ref. 3).

H_2 chemisorption experiments. Pd dispersion obtained from H_2 chemisorption was measured on an Autochem II 2920 chemisorption instrument by a HOT method (H_2-O_2 titration)[4]. Typically, ~50 mg of Pd catalyst was first treated at 165 °C with air for 4 h for fully oxide the surface Pd. After purging with high-purity He for 30 min, a 5% H_2/Ar mixture was injected into the reactor at 165 °C until H_2 adsorption was saturated. Assuming that one hydrogen molecule reduced one surface PdO to Pd and 0.5 hydrogen adsorbed on one Pd atom, the Pd dispersion was calculated as follows:

$$D_{Pd} = \frac{N_{surface}}{N_{total}} = \frac{\frac{2}{3}\text{amount of } H_2}{N_{total}} \times 100\%$$

H−D exchange. H−D exchange experiments were carried out in a flow quartz reactor at 22 °C (ref. 5). The formation rate of HD was measured by mass signal intensity (ion current). Before the test, the catalysts were heated in H_2 (10 mL min^{-1}) at 200 °C for 20 min. After cooling to room temperature, D_2 (10 mL min^{-1}) mixed with H_2 was passed through the sample. The gas hourly space velocity (GHSV) is 4.64×10^7 mL h^{-1} g_{metal}^{-1}. Under these conditions, the H−D exchange conversion were always kept below 15% for calculation of turnover frequency (TOF). Products (HD, H_2, and D_2)

were analysed with an online mass spectrometer (GAM200, InProcess Instruments). The mass/charge ratio (m/z) values used are 2 for H_2, 4 for D_2, and 3 for HD. The background HD exchanges from the corresponding support were deducted from the results.

Catalytic tests

Selective hydrogenation reactions. For 2-acetonaphthone, 2-naphthaldehyde, benzofuran, quinoline and furfural, the used dosage of catalysts and substrates are identical to acetophenone (AP) hydrogenation. Typically, a desired amount of the solid catalyst (2.5×10^{-3} mmol Pd) was placed in an ampule tube, followed by the addition of substrate (0.3 mmol) (S/C = 120) and 2 mL solvent. The ampule tube was loaded into the reactor. After the tube was purged six times with hydrogen, the final pressure was adjusted to 10 bar and the reactor was heated to 40 ^{o}C with vigorous stirring. After reaction the autoclave was cooled down to room temperature, the products were analyzed by a gas chromatograph (Agilent 7890A) equipped with an HP-5 column (30 m × 0.32 mm × 0.25 μm). For the substrates with much high activity, the substrate amount was raised to 8 mmol and S/C ratio was also increased for N-benzylidenemethylamine (S/C = 2000), nitrobenzene (S/C = 5000) and styrene hydrogenation (S/C = 5000), respectively. Conversion and selectivity were determined using n-tetradecane or n-decane as internal standards and the carbon balance of all the reactions is ~100%.

CHO and n-butylaldehyde hydrogenation. Specifically, CHO (0.07 mmol), catalyst (Pd 4 mmol%) and H_2O (2.0 mL) was added to a Teflon-lined steel autoclave. Before

each run, the autoclave was sealed and flushed with H_2 six times to remove the air. Then the autoclave was charged with 20 bar H_2, followed by heating from room temperature to 130 ºC within 5 min and kept at 130 ºC under vigorous stirring. After reaction the autoclave was cooled down to room temperature, the products were analyzed by a gas chromatograph (Agilent 7890A) equipped with an HP-5 column (30 m × 0.32 mm × 0.25 μm). For n-butylaldehyde hydrogenation, the reaction was carried out at 80 ºC, 20 bar H_2 and the products was analyzed by a gas chromatograph (Agilent 6890N) equipped with an HP-INNOWax column (30 m × 0.32 mm × 0.25 μ m). 1,4-Dioxane was used as the internal standard and the carbon balance of all the reactions is > 95%.

The calculation method for the catalytic performance

The conversion, selectivity to each product and the apparent TOF were defined as follows:

$$\text{Conversion} = \frac{(\text{mmols of reactants})_{in} - (\text{mmols of reactants})_{out}}{(\text{mmols of reactants})_{in}} \times 100\%$$

$$\text{Selectivity} = \frac{\text{mmols of product i}}{\sum \text{mmols of products}} \times 100\%$$

$$TOF/h^{-1} = \frac{\text{amt of substrate/mmol}}{\text{amt of}\frac{\text{Pd}}{\text{mmol}} \times Pd\ dispersion} \times \frac{\text{Conversion } (\sim 10\%)}{\text{t/h}}$$

Derivation of rate equations

We derive the kinetic expressions for the proposed mechanisms of AP hydrogenation in the main text based on the conventional Langmuir–Hinshelwood model with the following assumptions:

1) The AP molecules and hydrogen are adsorbed on different sites;

2) AP or intermediates concentration change results a negligible change in reaction rate.

In the following, the hydrogenation kinetic models of AP hydrogenation to phenyl ethanol involve adsorption, surface reaction and desorption as follows:

$$H_2(g) + 2S \overset{k_1}{\rightleftharpoons} 2HS \quad (1)$$

$$AP + S \overset{k_2}{\rightleftharpoons} APS \quad (2)$$

$$APS + HS \overset{k_3}{\rightarrow} APHS + S \quad (3)$$

$$APHS + HS \overset{k_4}{\rightarrow} PES + S \quad (4)$$

$$PES \overset{k_5}{\rightleftharpoons} PE + S \quad (5)$$

Where k_i is rate constant, and S represents empty Pd sites.

Assuming adsorption and desorption steps to be rate-determining does not lead to rate expressions consistent with the observed reaction orders.

When the first hydrogenation of AP is the rate-determining step (RDS):

$$r = \frac{k_3 K_{AP} C_{AP} \sqrt{P_{H2} K_{H2}}}{(1+K_{AP} C_{AP})(1+\sqrt{P_{H2} K_{H2}})} \quad (6)$$

When the second hydrogenation of AP is the RDS:

$$r = \frac{k_4 K_{APHS} K_{AP} C_{AP} (P_{H2} K_{H2})}{(1+K_{AP} C_{AP}+K_{APHS} K_{AP} C_{AP} \sqrt{P_{H2} K_{H2}})(1+\sqrt{P_{H2} K_{H2}})} \quad (7)$$

Where K_i is adsorption constant.

When the reactions are carried out under low H_2 pressure, the $1 + \sqrt{P_{H2} K_{H2}}$ term in the denominator is approximate to 1. Therefore, the reaction order based on H_2 at low H_2 pressure is half order for Eq. 6 and first order for Eq. 7, respectively.

Computational methods

We have employed the Vienna Ab Initio Package (VASP)[6, 7] to perform all the

density functional theory (DFT) calculations within the generalized gradient approximation (GGA) using the PBE[8] formulation. We have chosen the projected augmented wave (PAW) potentials[9, 10] to describe the ionic cores and take valence electrons into account using a plane wave basis set with a kinetic energy cutoff of 400 eV. Partial occupancies of the Kohn−Sham orbitals were allowed using the Gaussian smearing method and a width of 0.05 eV. The electronic energy was considered self-consistent when the energy change was smaller than 10^{-5} eV. A geometry optimization was considered convergent when the force change was smaller than 0.02 eV/Å. Grimme's DFT-D3 methodology[11] was used to describe the dispersion interactions.

The equilibrium lattice constants of hexagonal JDL P-6 unit cell were optimized, when using a 1×1×7 Monkhorst-Pack k-point grid for Brillouin zone sampling, to be a=37.617 Å, c=3.740 Å. We then use it to construct a (001) monolayer model with $p(1\times1)$ periodicity in the x and y directions and 1 stoichiometric layer in the z direction separated by a vacuum layer in the depth of 20 Å in order to separate the surface slab from its periodic duplicates. During structural optimizations, the gamma point in the Brillouin zone was used for k-point sampling, and all atoms were allowed to relax.

The equilibrium lattice constants of monoclinic CM COF unit cell were optimized, when using a 1×1×7 Monkhorst-Pack k-point grid for Brillouin zone sampling, to be a=35.503 Å, b=33.392 Å, c=4.034 Å, α=90°, β=75.6°, γ=90°. We then use it to construct a (001) monolayer model with $p(1\times1)$ periodicity in the x and y directions

and 1 stoichiometric layer in the z direction separated by a vacuum layer in the depth of 20 Å in order to separate the surface slab from its periodic duplicates. During structural optimizations, the gamma point in the Brillouin zone was used for k-point sampling, and all atoms were allowed to relax.

The adsorption energy (E_{ads}) of adsorbate A was defined as:

$$E_{ads} = E_{A/surf} – E_{surf} - E_{A(g)}$$

where $E_{A/surf}$, E_{surf} and $E_{A(g)}$ are the energy of adsorbate A adsorbed on the surface, the energy of clean surface, and the energy of isolated A molecule in a cubic periodic box with a side length of 20 Å and a 1×1×1 Monkhorst-Pack k-point grid for Brillouin zone sampling, respectively.

Finally, transition states (TS) for elementary reaction steps were determined by a combination of the nudged elastic band (NEB) method[12] and the dimer method[13-15]. In the NEB method, the path between the reactant and product is discretized into a series of structural images. The image that is closest to a likely TS structure was then employed as an initial guess structure for the dimer method.

The interaction energies between Py-COF/Be-COF and AP/CHO were carried out by using Gaussian 09 program suite[16]. All structures were optimized at the PBE0 level with Grimme's dispersion correction at the D3 level (PBE0-D3) by employing the def2-SVP basis set for all atoms (C, H, O, N). The interaction energies were calculated at the M06-2X/def2-SVP level by including basis set superposition errors at the PBE0-D3/def2-SVP optimized structures.

Supplementary Tables

Supplementary Table 1. Physical parameters and Pd 3*d* binding energies of Pd/COFs and commercial Pd/C.

Sample	BET surface area (m^2 g^{-1})	Pd dispersion (%)[a]	Pd dispersion (%)[b]	Pd $3d_{5/2}$ (eV)[c]	Pd^0/Pd^{2+} (%)[c]	L/B ratio[d]
Pd/Py-COF	567	26.2	21.3	335.3	68/32	0.14
Pd/Be-COF	1139	26.1	20.4	335.3	68/32	0.15
Pd/TB-COF	396	24.2	18.9	335.3	67/33	0.15
Pd/C	959	25.2	--	335.4	58/42	--

[a]Data obtained from CO chemisorption results. [b]Data obtained from H_2 chemisorption by a HOT method. [c]Data obtained from XPS results. [d]Calculated based on in situ FT-IR of CO adsorption.

Supplementary Table 2. The catalytic results of Pd/COFs and commercial Pd/C in AP hydrogenation.

Cat.	Conv. (%)	Sel. (%)		
		phenyl ethanol	ethylbenzene	ethylcyclohexane
Pd/Py-COF	>99	>99	0	0
Pd/Be-COF	30	>99	0	0
Pd/TB-COF	22	>99	0	0
Pd/C	>99	0	99	1

Reaction conditions: 40 °C, 10 bar of H_2, 2 mL of EtOH, 0.3 mmol of substrates, Pd catalysts 0.0.833 mol%, 3h.

Supplementary Table 3. The catalytic results of H-D exchange reaction over Pd NPs.

Cat.	Conv. (%)	TOF (h^{-1})	Normalized Activity
Pd/Py-COF	8.0	$3.7*10^6$	57
Pd/Be-COF	7.7	$3.6*10^6$	55
Pd/C	14.1	$6.5*10^6$	100

The reaction conditions see experimental section.

Supplementary Table 4. Synthesis of Py-COF under different conditions.[a]

Sample	Solvent (V: V: V)	State
1	O-dichlorobenzene: n-butanol: CH_3COOH = 10: 5: 1	Crystalline solid
2	O-dichlorobenzene: mesitylene: CH_3COOH: 5: 5: 1	Amorphous solid
3	THF: mesitylene: CH_3COOH: 5: 5: 1	Amorphous solid
4	1, 4-dioxane: mesitylene: CH_3COOH: 5: 5: 1	Amorphous solid
5[b]	O-dichlorobenzene: n-butanol: CH_3COOH = 10: 5: 1	Crystalline solid

[a]Reaction conditions: Py (57 mg), DMTA (40 mg), solvent (3.2 mL), 6 M CH_3COOH (0.2 mL), N_2 atmosphere, 85 °C, 3 d. [b]Py (285 mg), DMTA (200 mg), solvent (16 mL), 5 d.

Supplementary Table 5. Pd/Py-COF catalysed AP hydrogenation with different solvent.[a]

Solvent	Conv. (%)	Sel. (%)
EtOH	92	99
Isopropyl alcohol	69	99
H_2O	87 (22)[b]	99
CH_2Cl_2	50	99
Ethyl acetate	29	99
n-hexane	49	99
1, 4-Dioxane	20	99

[a]Reaction conditions: 40 °C, 10 bar of H_2, 2 mL of solvent, 0.3 mmol of AP, Pd catalysts 0.833mol%, 2h. [b]The parentheses refers to Pd/Be-COF.

Supplementary Figures

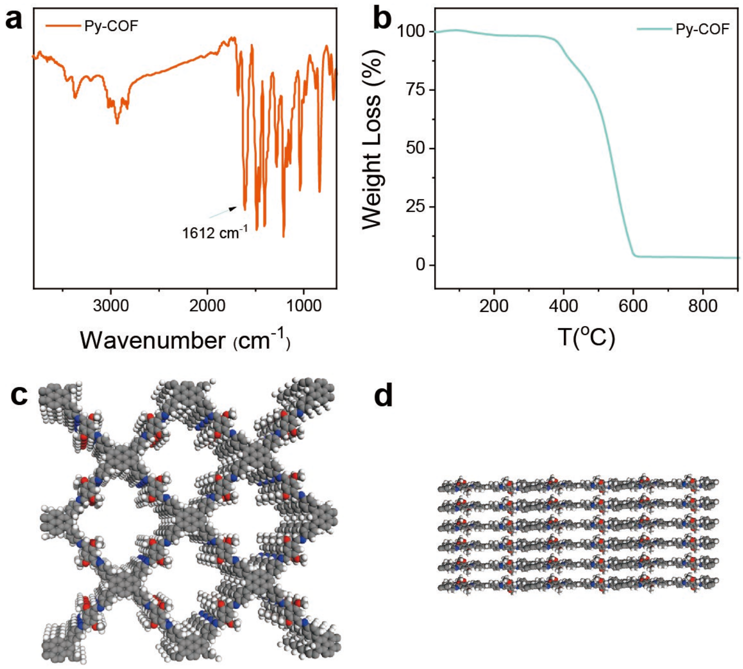

Supplementary Fig. 1 a FTIR spectrum, **b** TGA results, and **c** top and **d** side views of the energy-minimized models of Py-COF.

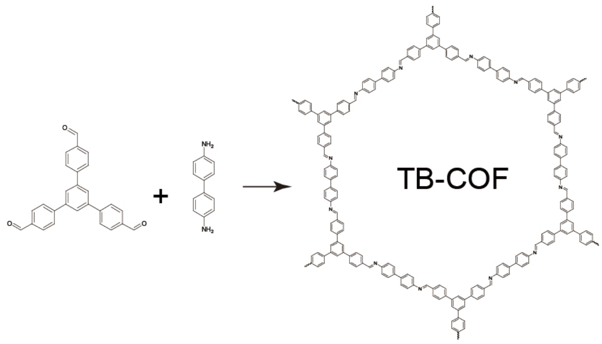

Supplementary Fig. 2 Synthesis of TB-COF.

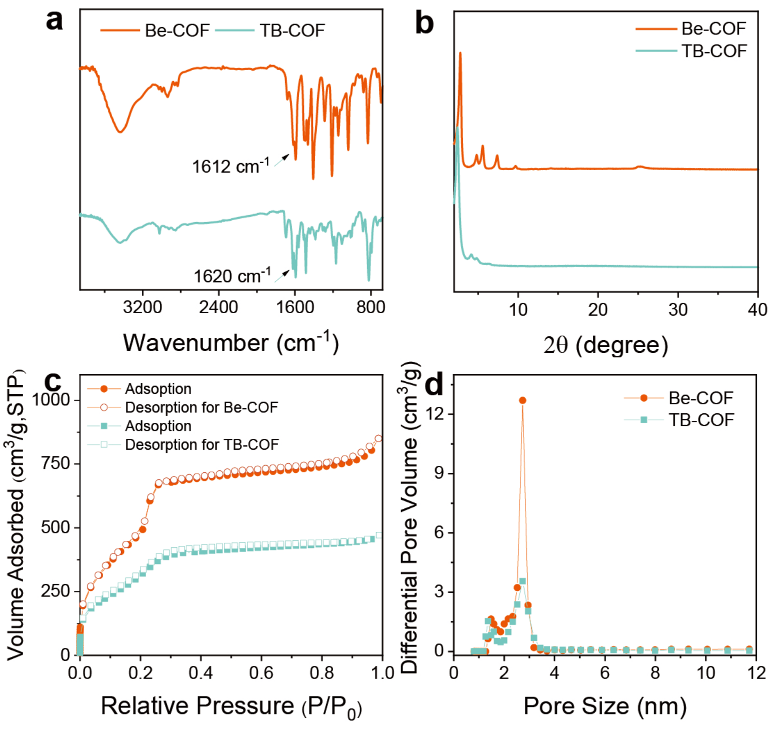

Supplementary Fig. 3 **a** FT-IR spectra, **b** PXRD patterns, **c** N_2 adsorption isotherms and **d** pore size distribution of Be-COF and TB-COF.

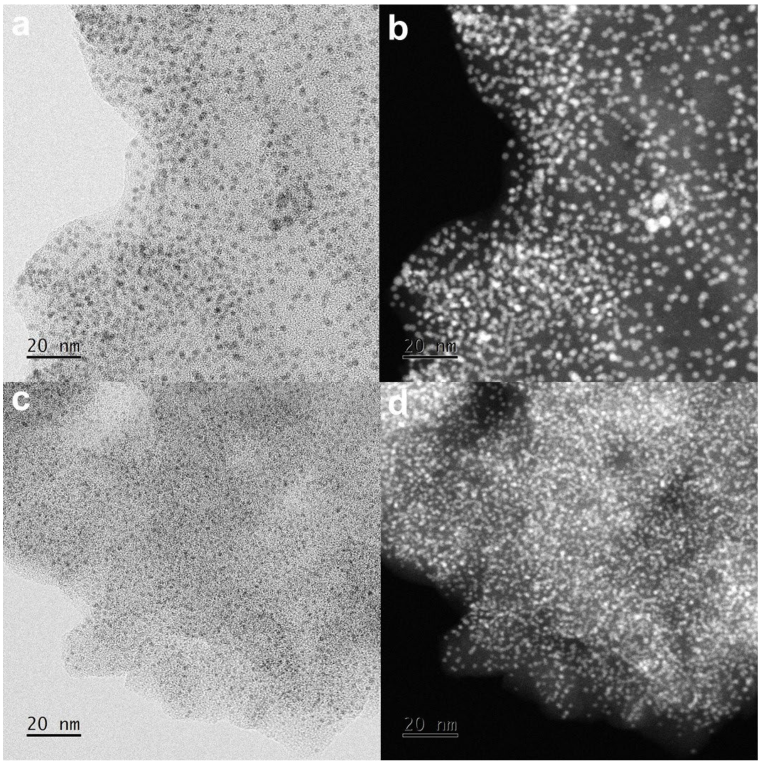

Supplementary Fig. 4 **a** HRTEM image and **b** HAADF-STEM image of Pd/TB-COF. **c** HRTEM image and **d** HAADF-STEM image of Pd/Py-COF.

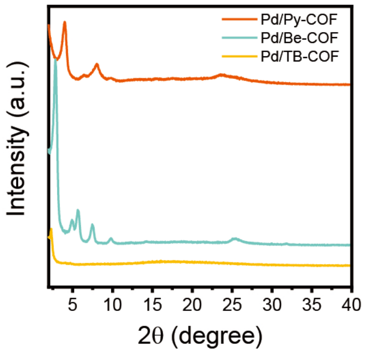

Supplementary Fig. 5 PXRD patterns of Pd/Py-COF, Pd/Be-COF and Pd/TB-COF.

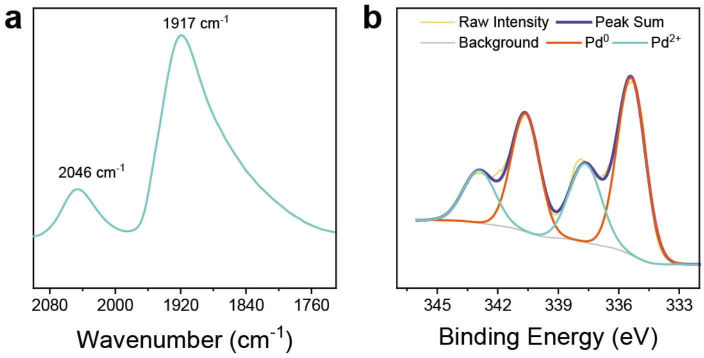

Supplementary Fig. 6 a In situ FTIR of CO adsorption and **b** Pd 3*d* XPS core level spectra of Pd/TB-COF.

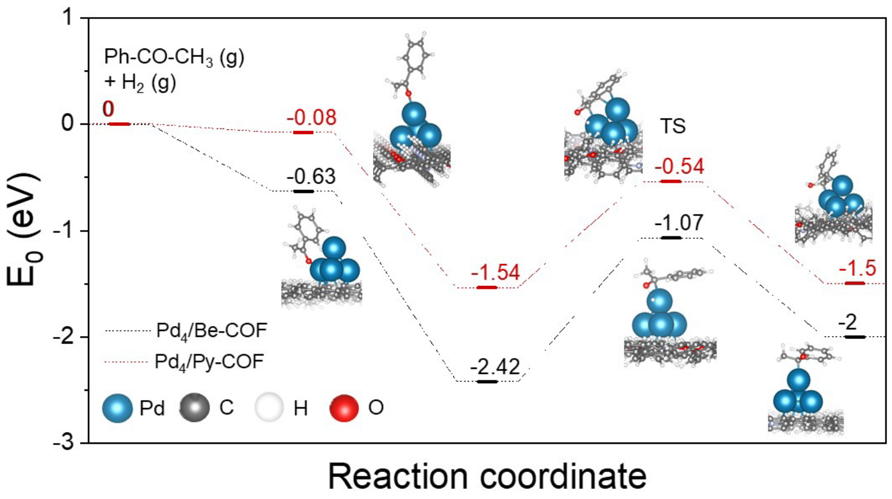

Supplementary Fig. 7 Calculated energy profile of the O hydrogenation step of AP hydrogenation on Pd_4/Be-COF and Pd_4/Py-COF.

Noting: The catalytic reaction begins with AP adsorption on an ensemble of vacant Pd sites as chemisorbed AP. Meanwhile, H_2 dissociated adsorb on a Pd site pair, forming two adsorbed hydrogen adatoms (H*). The reactive H* first attack the O atom, forming an O-H bond, followed by the second H* attack to the carbonyl C. Since the O addition step is kinetically relevant, the O hydrogenation step was calculated. Because of long distance of aromatic group from the reactant in our Pd_4 models, the π-π interaction promoted AP hydrogenation can not be clearly reflected from the DFT calculation. However, the DFT calculation results show that the O hydrogenation step of AP hydrogenation could be significantly decreased via the interaction between phenyl ring and Pd atom. On the basis of our control experiments and the strong interaction between the benzene ring of AP molecule with pyrene rings around Pd surface, we can conclude that the Pd NPs confined in the Py-COF could accelerate the AP hydrogenation activity via π-π interaction.

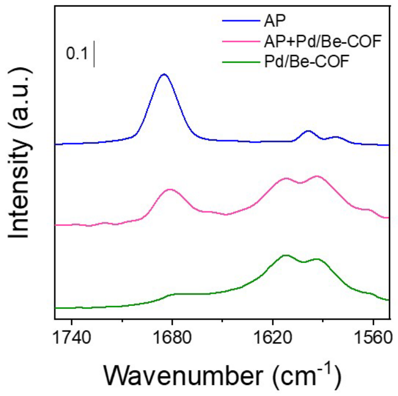

Supplementary Fig. 8 FT-IR spectra of AP, Pd/Be-COF and AP adsorbed on Pd/Be-COF.

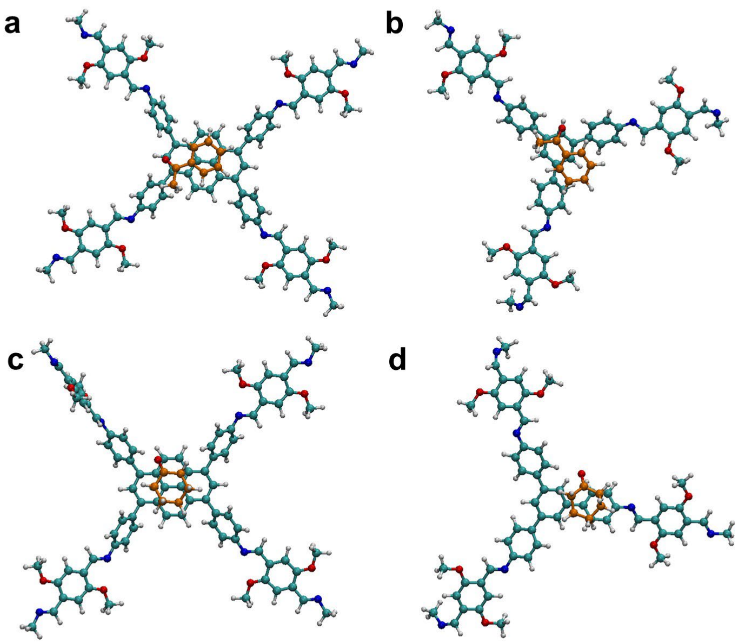

Supplementary Fig. 9 The models for the calculated interactions energies by DFT. **a** Py-COF/AP, **b** Be-COF/AP, **c** Py-COF/CHO and **d** Be-COF/CHO.

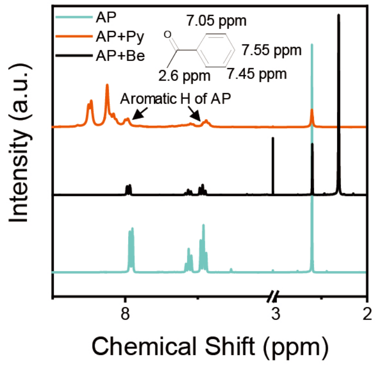

Supplementary Fig. 10 ^{1}H-NMR spectra of AP, AP + mesitylene (molar ratio: 1:2) and (iii) AP + pyrene (molar ratio: 1:2).

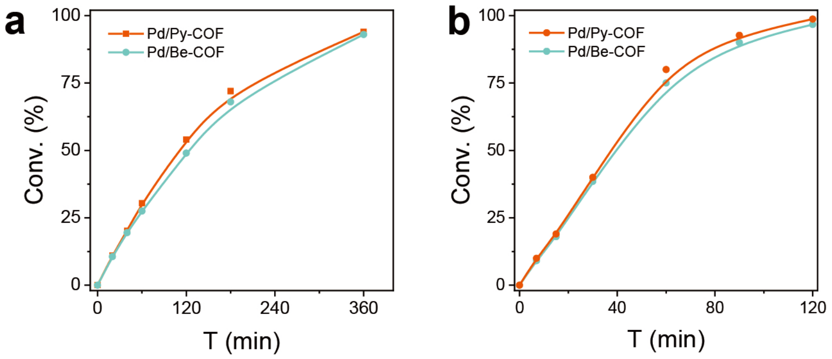

Supplementary Fig. 11 Reaction profiles of supported Pd NPs in **a** CHO hydrogenation (130 °C, 20 bar of H_2, 0.07 mmol of CHO, Pd catalysts 4 mmol%, 2 mL of H_2O) and **b** n-butyraldehyde hydrogenation (80 °C, 20 bar of H_2, 0.07 mmol of butyraldehyde, Pd catalysts 4 mmol%, 2 mL H_2O).

Noting: For CHO hydrogenation, almost no activity was observed on Pd/COFs using EtOH as the solvent due to the poison effect of EtOH to Pd[17]. According to the previous report[18], H_2O could boost the C=O hydrogenation rate through the solvation of sorbed hydrogen[19]. Therefore, the kinetic curves of CHO hydrogenation on Pd/COFs were carried out with H_2O as the solvent. As shown in the Supplementary Fig. 11a, the almost coincided kinetic curves of CHO hydrogenation suggest Pd/COFs have similar catalytic ability to CHO hydrogenation. Similar trends were also obtained using n-butyraldehyde as the substrates (Supplementary Fig. 11b). Notably, Pd/Py-COF shows higher activity than Pd/Be-COF (87% versus 22%) in AP hydrogenation with H_2O as solvent (Supplementary Table 5), showing that H_2O does not alter the AP activity order of Pd/COFs.

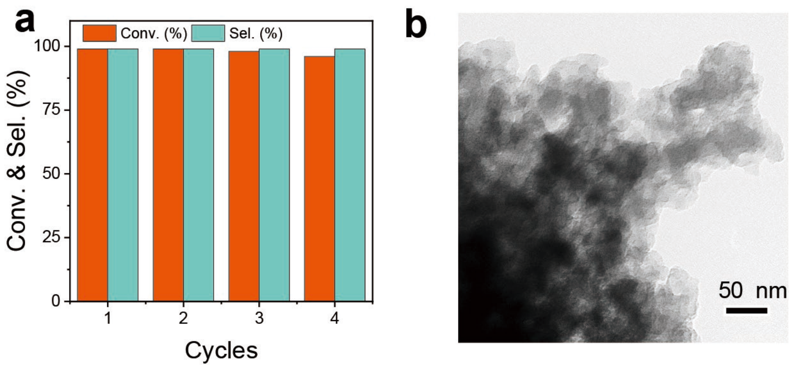

Supplementary Fig. 12 a Recycling stability of Pd/Py-COF in AP hydrogenation, **b** TEM image of Pd/Py-COF after the fourth cycle (Reaction conditions: 40 °C, 10 bar of H_2, 2 mL of EtOH, 0.3 mmol of substrates, Pd catalysts 0.0.833 mol%, 3h.)

Supplementary References

1. Ascherl, L., Evans, E. W., Hennemann, M., Di Nuzzo, D., Hufnagel, A. G., Beetz, M., Friend, R. H., Clark, T., Bein, T. & Auras, F. Solvatochromic covalent organic frameworks. *Nat. Commun.* **9**, 1-8 (2018).
2. Kuhnert, N., Rossignolo, G. M., & Lopez-Periago, A. The synthesis of trianglimines: on the scope and limitations of the [3+3] cyclocondensation reaction between (1R, 2R)-diaminocyclohexane and aromatic dicarboxaldehydes. *Org. Biomol. Chem.* **1**, 1157-1170 (2003).
3. Canton, P., Fagherazzi, G., Battagliarin, M., Menegazzo, F., Pinna, F., & Pernicone, N. Pd/CO average chemisorption stoichiometry in highly dispersed supported Pd/γ-Al_2O_3 catalysts. *Langmuir* **18**, 6530-6535 (2002).
4. Su, S. C., Carstens, J. N., & Bell, A. T. A study of the dynamics of Pd oxidation and PdO reduction by H_2 and CH_4. *J. Catal.* **176**, 125-135 (1998).
5. Guo, M., Peng, J., Yang, Q., & Li, C. Highly active and selective RuPd bimetallic NPs for the cleavage of the diphenyl ether C–O bond. *ACS Catal.* **8**, 11174-11183 (2018).
6. Kresse, G. & Furthmüller, J. Efficiency of Ab-initio total energy calculations for metals and semiconductors using a plane-wave basis set. *Comput. Mater. Sci.* **6**, 15−50 (1996).
7. Kresse, G. & Furthmüller, J. Efficient lterative schemes for Ab initio total-energy calculations using a plane-wave basis set. *Phys. Rev. B* **54**, 11169−11186 (1996).
8. Perdew, J. P., Burke, K. & Ernzerhof, M. Generalized gradient approximation made simple. *Phys. Rev. Lett.* **77**, 3865−3868 (1996).

9. Kresse, G. & Joubert, D. From ultrasoft pseudopotentials to the projector augmented-wave method. *Phys. Rev. B* **59**, 1758-1775 (1999).

10. Blöchl, P. E. Projector augmented-wave method. *Phys. Rev. B* **50**, 17953−17979 (1994).

11. Grimme, S., Antony, J., Ehrlich, S. & Krieg, H. *J. Chem. Phys.* **132**, 154104 (2010).

12. Henkelman, G., Uberuaga, B. P. & Jonsson, H. *J. Chem. Phys.* **113**, 9901 (2000).

13. Henkelman, G. & Jonsson, H. *J. Chem. Phys.* **111**, 7010–7022 (1999).

14. Olsen, R. A., Kroes, G. J., Henkelman, G., Arnaldsson, A. & Jonsson, H. *J. Chem. Phys.* **121** 9776–9792 (2004).

15. Heyden, A., Bell, A. T. & Keil, F. J. *J. Chem. Phys.* **123**, 224101 (2005).

16. Frisch, M. J.; Trucks, G. W.; Schlegel, H. B.; Scuseria, G. E.; Robb, M. A.; Cheeseman, J. R.; Scalmani, G.; Barone, V.; Mennucci, B.; Petersson, G. A.; Nakatsuji, H.; Caricato, M.; Li, X.; Hratchian, H. P.; Izmaylov, A. F.; Bloino, J.; Zheng, G.; Sonnenberg, J. L.; Hada, M.; Ehara, M.; Toyota, K.; Fukuda, R.; Hasegawa, J.; Ishida, M.; Nakajima, T.; Honda, Y.; Kitao, O.; Nakai, H.; Vreven, T.; Montgomery, J. A.; Peralta, Jr, J. E.; Ogliaro, F.; Bearpark, M.; Heyd, J. J.; Brothers, E.; Kudin, K. N.; Staroverov, V. N.; Keith, T.; Kobayashi, R.; Normand, J.; Raghavachari, K.; Rendell, A.; Burant, J. C.; Iyengar, S. S.; Tomasi, J.;. Cossi, M; Rega, N.; Millam, J. M.; Klene, M.; Knox, J. E.; Cross, J. B.; Bakken, V.; Adamo, C.; Jaramillo, J.; Gomperts, R.; Stratmann, R. E.; Yazyev, O.; Austin, A. J.; Cammi, R.; Pomelli, C.; Ochterski, J. W.; Martin, R. L.; Morokuma, K.; Zakrzewski, G. A. Voth, P. Salvador, J. J. Dannenberg, S. Dapprich, A. D. Daniels, O. Farkas, J. B. Foresman, V. G.; Ortiz, J. V.; Cioslowski, J.; Fox, D. J. Revision A. 02; 2009.

17. Li, X., Cheng, L. & Wang, X. Selective phenol hydrogenation under mild condition over Pd catalysts supported on Al_2O_3 and SiO_2. *Res. Chem. Intermediat.* **45**, 1249-1262 (2019).

18. He, J., Zhao, C. & Lercher, J. A. Impact of solvent for individual steps of phenol hydrodeoxygenation with Pd/C and HZSM-5 as catalysts. *J. Catal.* **309**, 362-375 (2014).

19. Cheng, G., Jentys, A., Gutiérrez, O. Y., Liu, Y., Chin, Y. H., & Lercher, J. A. Critical role of solvent-modulated hydrogen-binding strength in the catalytic hydrogenation of benzaldehyde on palladium. *Nat. Catal.* **4**, 976-985 (2021).
